# puma 3.0: improved uncertainty propagation methods for gene and transcript expression analysis

**DOI:** 10.1186/1471-2105-14-39

**Published:** 2013-02-05

**Authors:** Xuejun Liu, Zhenzhu Gao, Li Zhang, Magnus Rattray

**Affiliations:** 1 College of Computer Science and Technology, Nanjing University of Aeronautics and Astronautics, 29 Yudao St., Nanjing, 210016, China; 2 Faculty of Life Sciences, University of Manchester, Oxford Road, Manchester M13 9PT, UK

## Abstract

**Background:**

Microarrays have been a popular tool for gene expression profiling at genome-scale for over a decade due to the low cost, short turn-around time, excellent quantitative accuracy and ease of data generation. The Bioconductor package *puma* incorporates a suite of analysis methods for determining uncertainties from Affymetrix GeneChip data and propagating these uncertainties to downstream analysis. As isoform level expression profiling receives more and more interest within genomics in recent years, exon microarray technology offers an important tool to quantify expression level of the majority of exons and enables the possibility of measuring isoform level expression. However, *puma* does not include methods for the analysis of exon array data. Moreover, the current expression summarisation method for Affymetrix 3’ GeneChip data suffers from instability for low expression genes. For the downstream analysis, the method for differential expression detection is computationally intensive and the original expression clustering method does not consider the variance across the replicated technical and biological measurements. It is therefore necessary to develop improved uncertainty propagation methods for gene and transcript expression analysis.

**Results:**

We extend the previously developed Bioconductor package *puma* with a new method especially designed for GeneChip Exon arrays and a set of improved downstream approaches. The improvements include: (i) a new gamma model for exon arrays which calculates isoform and gene expression measurements and a level of uncertainty associated with the estimates, using the multi-mappings between probes, isoforms and genes, (ii) a variant of the existing approach for the probe-level analysis of Affymetrix 3’ GeneChip data to produce more stable gene expression estimates, (iii) an improved method for detecting differential expression which is computationally more efficient than the existing approach in the package and (iv) an improved method for robust model-based clustering of gene expression, which takes technical and biological replicate information into consideration.

**Conclusions:**

With the extensions and improvements, the *puma* package is now applicable to the analysis of both Affymetrix 3’ GeneChips and Exon arrays for gene and isoform expression estimation. It propagates the uncertainty of expression measurements into more efficient and comprehensive downstream analysis at both gene and isoform level. Downstream methods are also applicable to other expression quantification platforms, such as RNA-Seq, when uncertainty information is available from expression measurements. *puma* is available through Bioconductor and can be found at http://www.bioconductor.org.

## Background

Microarrays have been applied to high-throughput gene expression profiling for over a decade due to several advantages, e.g. high coverage, low cost, short turn-around time, excellent quantitative accuracy and ease of data generation. It has been shown recently that microarrays still remain an efficient and reliable tool for expression quantification especially for low-abundance targets [1]. We previously developed the Bioconductor package *puma*[2] for Affymetrix GeneChip data analysis. In the initial probe-level analysis, *puma* uses the multi-mgMOS method [3] to obtain an expression estimate for each gene and a level of uncertainty associated with this estimate. In the downstream analysis, *puma* propagates these uncertainties to principal component analysis, differential expression detection and gene expression clustering using methods NPPCA [4], PPLR [5] and PUMA-CLUST [6], respectively, and obtains improved analysis results. In addition to expression measurements obtained from microarrays, these downstream methods are also applicable to other expression quantification platforms, e.g. RNA-Seq based on high throughput sequencing technology, providing a level of uncertainty is associated with each measurement.

As the analysis of alternative splicing gains more and more interest in recent years, exon microarray technology, such as Affymetrix GeneChip Exon arrays, provides an option for measuring isoform level expression. It is therefore necessary for *puma* to include methods for propagating isoform expression uncertainty in the analysis of exon array data. Furthermore, the current probe-level analysis method, multi-mgMOS, obtains unstable expression estimates for low expression genes which can adversely affect the downstream analysis results. For the downstream analysis, the PPLR method for differential expression detection is computationally expensive and the PUMA-CLUST method for expression clustering does not consider the variance across the replicated technical and biological measurements. For all these reasons, we present here a new version of the *puma* package which incorporates a suite of improved probe-level analysis methods for gene and transcript expression summarisation and uncertainty propagation methods for the downstream analysis. The new version of the package covers the wide range of quantitative expression analysis of microarray at both gene and isoform level with the great benefit from propagating uncertainty associated with expression estimates into various advanced downstream analyses.

Affymetrix microarrays use 25-base long probes to measure transcript abundance. Traditional 3’ GeneChips use two types of probes, perfect match (PM) and mismatch (MM) probes. A PM probe matches the target sequence exactly, whereas the corresponding MM probe differs from the PM probe in the middle base which is changed to the complementary one. MM probes are introduced to act as a control for cross hybridisation and other types of background signal. The GeneChip Exon arrays use only PM probes to obtain higher density of coverage and make exon, isoform and gene level profiling possible. Many probe-level analysis methods for 3’ arrays such as PLIER [7] and RMA [8] which do not use MM probe intensities, can be applied to exon arrays directly for exon or gene level expression calculation by using probe-to-exon or probe-to-gene mappings, respectively. With the estimated exon and gene expression, it is possible to perform alternative splicing detection by measuring exon-gene expression ratios [9-11]. In addition to calculating exon and gene expression ratios, isoform expression levels can also be quantified for a more refined downstream analysis.

The expression calculation at isoform level is non-trivial since one probe can be mapped to multiple transcripts or gene loci [12]. Also, an important characteristic of Affymetrix microarray probes is that they have different sensitivity to transcript abundance according to their sequence content. Many probe-level analysis approaches for 3’ arrays account for these probe-specific effects and have obtained improved results [3,13]. Moreover, a level of uncertainty associated with estimated isoform expression would help downstream analyses to obtain more biologically relevant results. With available multi-mappings between probes and Ensembl transcripts, some methods have recently been proposed to address the expression calculation for known isoforms, such as MMBGX [14] and MEAP [15]. MMBGX uses a hierarchical Bayesian model to calculates the expression level of target transcripts and results in a posterior distribution of each isoform expression. MMBGX is solved by MCMC method and is therefore computationally intensive. After background removal, MEAP adopts a non-negative matrix factorisation approach to summarise isoform expression as a point estimate and does not provide a level of uncertainty associated with this estimate. MMBGX and MEAP perform cross-hybridisation correction according to different GC content for probes, removing probe-specific effects to a certain extent. However, it has been shown that specific hybridisation also presents probe-specific variations [8,16]. We developed a new gamma model for exon array data (GME), which accounts for probe-effects in specific hybridisation and multi-mappings between probes, transcripts and genes. The GME model parameters are estimated by Maximum a Posteriori (MAP) optimisation to give isoform and gene level expression measurements with a level of uncertainty of these estimates, provided by a MAP-Laplace approximation [17]. The new method has been implemented as an R function in the new version of the *puma* package.

For traditional 3’ GeneChips, PM probes are thought to mainly measure specific hybridisation and MM probes measure non-specific hybridisation and other background. However, probes for low expression genes often obtain higher background than true signal. When combining PM and the corresponding MM probe intensities to calculate gene expression, the resulting gene expression measurements can be unstable for low expression genes, especially on a log scale. For this reason, most popular methods provide an option of using PM probes only in order to obtain more stable expression values on the log scale, such as PLIER [7], dCHIP [16] and RMA [8]. The previous method for 3’ GeneChips in *puma*, multi-mgMOS [3], combines both PM and MM probe intensities to calculate gene expression values and provide a level of uncertainty associated with the measurements. For low expression genes the estimated logarithmic expression values are usually negative and the associated variance is typically large. These expression measurements with large error can further affect downstream analyses and may lead to incorrect biological conclusions. This is especially the case when the mean expression estimates are processed by methods outside of the *puma* package which do not account for measurement uncertainty. To alleviate this problem, we propose PM-only multi-mgMOS for 3’ arrays, which uses only PM probe intensities and obtains more stable gene expression estimates for low expression genes.

For the downstream analyses of gene expression, the new version of *puma* includes two newly improved approaches for finding differentially expressed (DE) genes and gene expression clustering. The previous method PPLR for finding DE genes considers the probe-level measurement error, which can improve results when there are few replicates available [5,18]. PPLR uses an importance sampling procedure in the variational EM solver which leads to computational inefficiency since the number of samples needs to be increased to gain better accuracy. By adding a layer of hidden variables to the hierarchical Bayesian model, inference in the PPLR model is faster due to the elimination of this inefficient importance sampling step [19]. The PUMA-CLUST method provided by the previous version of *puma* propagates probe-level uncertainty to improve results of standard Gaussian mixture clustering of gene expression [6]. The recently proposed PUMA-CLUSTII [20] approach improves PUMA-CLUST in several aspects. First, variance across the replicated technical and biological measurements for the same experimental condition is considered. Second, a Student’s *t*-distribution is adopted as the clustering components to improve the robustness of the method. Finally, the optimal number of components can be automatically found, and this is especially important for the clustering when the ground truth in the data is unknown.

## Implementation

### Extended and improved function components in puma

**Figure 1:**
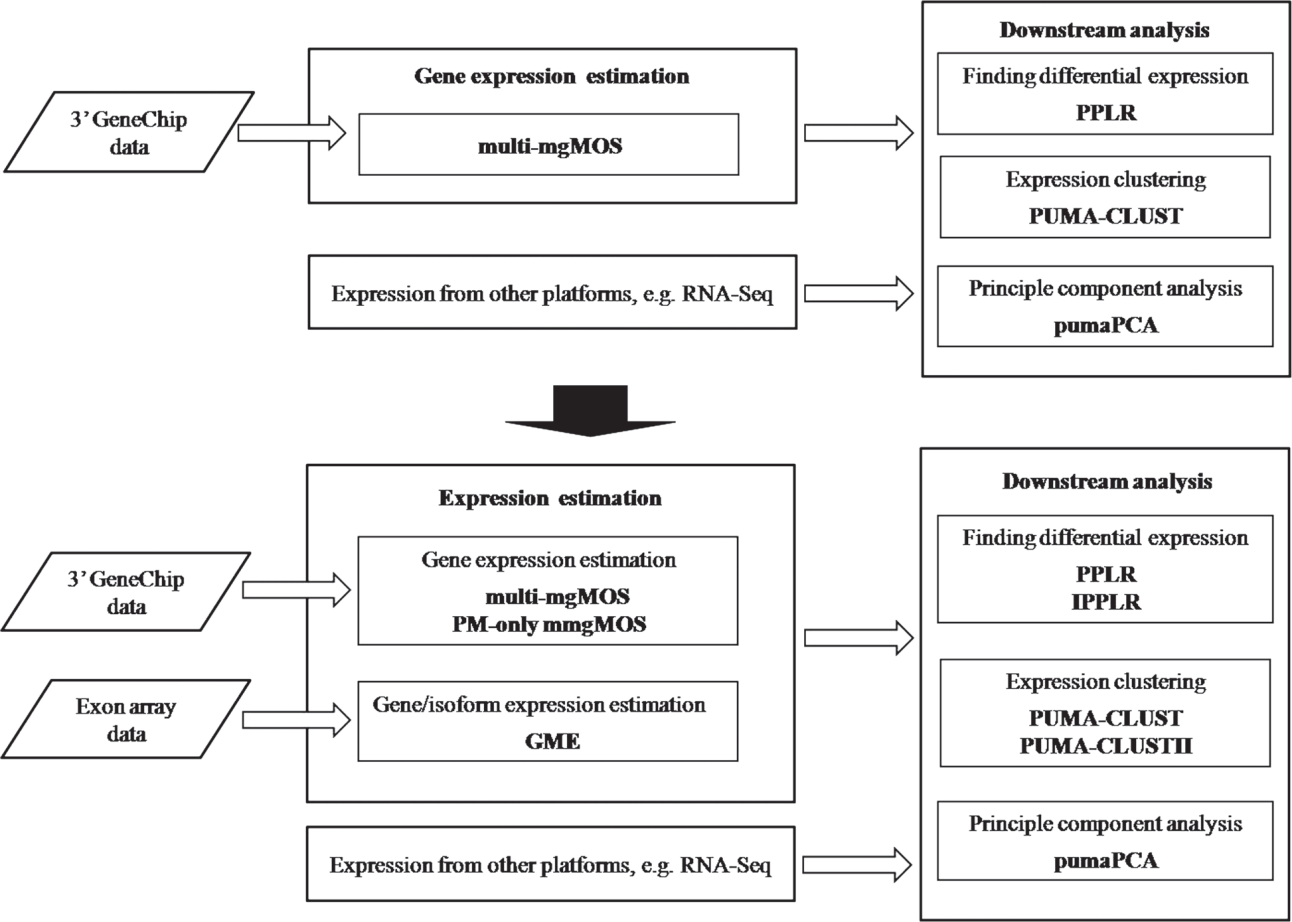
**Function components of the previous and new version of puma.** The upper part of the figure shows the function components of the previous version of *puma* package and the lower part shows the new version. After the extension and improvement, the new version covers expression analysis for 3’ GeneChip and Exon array data at both gene and isoform level.

*puma* includes two levels of analyses for expression data, expression summarisation and downstream analyses. At the summarisation level of analysis, the previous version of *puma* as described in [2] can only processe 3’ GeneChip data using mainly multi-mgMOS. With the obtained gene expression measurements and the associated measurement uncertainty from microarrays or other platforms, *puma* propagates uncertainty into the downstream analyses, including PPLR for finding DE genes, PUMA-CLUST for gene expression clustering and NPPCA [4] for principal component analysis of gene expression. The diagram of function components for the previous *puma* is shown in the upper part of Figure 1. After the extension and improvement in this paper, the functions of the new version of *puma* are illustrated in the lower part of Figure 1. The new version provides the following contributions: 

•GME - In addition to traditional 3’ GeneChip data, the new version is capable of processing Exon array data using a new model GME at the summarisation level of analysis. From the Exon array data analysis, both gene and isoform expression can be computed.

•PM-only multi-mgMOS - PM-only multi-mgMOS is included to improve the stability of multi-mgMOS for gene expression estimation.

•IPPLR - At the downstream analyses, the new version of the package contains IPPLR as an improvement to speed up PPLR for detecting differential expression.

•PUMA-CLUSTII - For expression clustering, PUMA-CLUSTII is introduced to consider the technical and biological variance across experimental replicates. The new clustering method increases the robustness of clustering and automatically selects the optimal number of clusters by model selection.

With these contributions, methods in *puma* can process both gene and isoform expression, making *puma* useful in the analysis of alternative splicing. See Methods for more details on these algorithms.

### Multi-mappings between probes and isoforms

The increasing availability of mappings of microarray probes to isoforms in the Ensembl database can be used to perform isoform expression estimation. In particular, multi-mappings between probes and isoforms are helpful in separating the intensity contributions from probes shared by multiple isoforms. Transcript expression estimation may benefit from this intensity separation. The database GATExplorer [12] integrates information from multiple biological sources (including Ensembl database and probe sequences of Affymetrix microarrays) to provide the mappings between microarray probes and the functional transcriptional entities, i.e. gene loci, transcripts, exons and ncRNAs. We include the multi-mappings between Exon array probes, isoforms and genes obtained from GATExplorer into the separate Bioconductor data package *pumadata* which contains example and annotation data used by *puma*. Mappings for human, mouse and rat exon arrays are included and this makes *puma* applicable to all types of Affymetrix Exon arrays.

### Using the extended functions in puma

The new version of *puma* and the related *pumadata* package can be found at http://www.bioconductor.org. The GEM model is implemented in the function gmoExon to calculate gene and isoform level expression for Exon arrays. The PM-only multi-mgMOS method is implemented in the function PMmmgmos to estimate stable gene expression for Affymetrix GeneChips. The improved PPLR for detecting DE genes is implemented in the function pumaCombImproved. The PUMA-CLUSTII is implemented in the function pumaclustii for robust expression clustering. To use these functions, type library(puma) and library(pumadata) at R prompt to load *puma* package and the data package. A quick start of each of these functions is described below. For detailed use of these functions, please refer to the user manual of the *puma* package.

#### Gamma model for Exon arrays

The expression summarisation method for Exon arrays is GME. The method makes use of multi-mappings between probes, isoforms and genes obtained from GATExplorer to aid the calculation of gene and isoform expression. The mappings are included in the individual package *pumadata*. The following code shows a quick start of this method.

##### 

The above code loads exon array data (CEL files) in the working directory as an AffyBatch object and processes it using GME method. Among the parameters, exontype can be one of “Human”, “Mouse” and “Rat”, indicating the exon chip type. GT can be one of “gene” and “transcript”, specifying the expression estimated at gene and isoform level, respectively. gsnorm specifies the algorithm used by the global scaling normalisation and can be one of “mean”, “median”, “meanlog” and “none”. “mean” and “meanlog” are mean-centered normalisation on raw and the log scale, respectively, “median” is median-centered normalisation and “none” means no global scaling normalisation. The value of gmoExon is an object of class exprReslt which stores the estimated expression and a level of uncertainty associated with this measurement.

#### PM-only multi-mgMOS for Affymetrix GeneChips

PM-only multi-mgMOS increases the stability of the original multi-mgMOS method, especially for weakly expressed genes. We use an example dataset included in the *pumadata* package to demonstrate the use of this method.

##### 

The first parameter of the function PMmmgmos is an AffyBatch object containing the raw probe intensities. The parameter gsnorm has the same meaning as that in the function gmoExon. The value of PMmmgmos is an object of class exprReslt which contains the estimated gene expression and the corresponding estimation uncertainty.

#### Improved PPLR for finding DE genes

IPPLR is designed to improve the computational efficiency of the original PPLR for finding differential expression. Similar to PPLR, it includes two steps to detect DE genes. At the first step, the function pumaCombImproved is used to combine expression from replicates to give a single measurement for the related condition. At the second step, the existing function pumaDE is used to calculate the PPLR (probability of positive log-ratio) values to identify DE genes. We use an example dataset in the *puma* package to demonstrate the use of this method as below.

##### 

The parameter of pumaCombImproved is an object of class ExpressionSet and can also be the outputs from GME, PM-only multi-mgMOS or multi-mgMOS. The function pumaDE generates lists of genes ranked by the PPLR values which indicate the significance of differential expression.

#### PUMA-CLUSTII for robust clustering

The existing clustering method PUMA-CLUST in *puma* considers uncertainty of gene expression but does not take into account the technical and biological variance when replicates are available. PUMA-CLUSTII is proposed to address this problem. It also adopts more robust components by using a Student’s *t* distribution instead of the Gaussian components used by PUMA-CLUST. We use an example dataset in the *puma* package to show the use of this method.

##### 

The first two parameters of pumaClustii are data frames containing the expression measurements and the associated uncertainty respectively. The minimum and maximum numbers of clusters are specified by the parameters mincls and maxcls, respectively. The parameter conds indicates the number of conditions involved in the data and reps is a vector specifying which condition each column of the input data frame belongs to. The result is a list containing the center of clustering components, the membership of components for each data point, the optional number of clusters and other auxiliary information.

## Results and discussion

### Datasets

#### MAQC dataset

We use the well studied Microarray Quality Control (MAQC) dataset [21] to evaluate most of the extensions of the new version of *puma* at gene expression level. MAQC project measured gene expression levels from high-quality RNA samples to assess the comparability across multiple platforms. We select two RNA samples, the universal human reference RNA (UHRR) and the human brain reference RNA (HBRR), from Affymetrix Exon array and Affymetrix U133 GeneChip platforms. Each sample type has five replicates for both platforms. Experiments of Exon arrays were carried out in two independent labs: McGill University (MU) and Virginia Tech (VT). We randomly selected data from MU for the evaluation of GME. For U133 GeneChips, we use data AFX_1_[A-B][1-5] from GSE5350. Apart from microarray experiments, MAQC project also conducted qRT-PCR experiments for around one thousand genes which can be served as a gold-standard to benchmark gene expression values estimated from other platforms [22,23].

Among the qRT-PCR data, we use the method similar to [23] to filter out DE and non-DE genes with high certainty. Firstly, we select genes which were found to be “present” for at least three qRT-PCR replicate assays. Secondly, average gene expression over replicates is calculated for each sample. Genes with absolute log-ratio between the UHRR and HBRR samples less than 0.2 are taken as “non-DE” genes. Those with log-ratio greater than 2.0 are “DE+” genes which are up-regulated in UHRR sample and those with log-ratio less than -2.0 are “DE-” genes being down-regulated in UHRR sample. Finally, we map these non-DE and DE genes to Exon array and U133 GeneChip platforms and obtain the corresponding mapped genes and probe-sets for each platform as shown in Table 1. Using these qRT-PCR validated data, we produce receiver operator characteristic (ROC) curves for various combinations of gene expression estimation methods and DE gene detection methods with the consideration of the direction sign of regulation.

**Table 1:** Number of qRT-PCR validated non-DE and DE genes and probe-sets for Exon arrays and H133 GeneChips

	**non-DE**	**DE**	
		**DE+**	**DE-**
Exon arrays	87	116	102
U133 GeneChips	204	185	267

Non-DE and DE genes obtained from qRT-PCR data with high certainty are mapped to Exon arrays and Affymetrix U133 GeneChips. Exon arrays obtain 305 corresponding genes and U133 GeneChips contain 656 related probe-sets. The symbols “+” and “-” stand for up- and down-regulation in UHRR, respectively.

#### HNSCC dataset

The qRT-PCR validated head and neck squamous cell carcinoma (HNSCC) dataset [15] is used to verify the isoform expression calculated by GME. In HNSCC dataset, 15 cell lines from tongue and larynx were cultured and samples were assayed using Affymetrix Human Exon 1.0 ST microarrays. Amplification of the chromosome region 11q13 is a common genomic alteration in HNSCC. The 15 cell lines are divided into two sample groups, with 11q13 amplification (11q13+) and without 11q13 amplification (11q13-). 11q13+ group contains seven cell lines and 11q13- group contains eight. qRT-PCR experiments were performed for four alternatively spliced variants of two genes (ORAOV1 and NEO1) located in the 11q13 amplified region and associated with HNSCC. We use GME to calculate the expression levels for the four isoforms in all 15 cell lines and then apply PPLR to identify the differential expressed transcripts (DETs). The detected DETs are compared with qRT-PCR findings to verify the performance of GME.

### Accuracy of gene expression estimation for Exon array data

**Figure 2:**
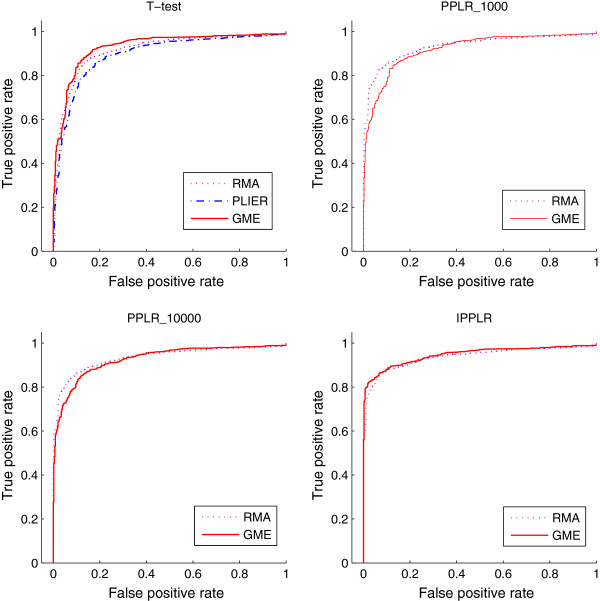
**ROC curves from different methods for 2-replicate Exon array data.** The ROC curves are obtained from the average over the 5 runs each of which randomly selects two replicates. Gene expression estimation methods RMA, PLIER and GMA, are combined with different finding-DE-gene methods, *t*-test, PPLR and IPPLR. PLIER provides only a point estimate for gene expression and therefore is not applicable to PPLR and IPPLR. The number after PPLR indicates the sample number used in the importance sampling of the algorithm.

**Figure 3:**
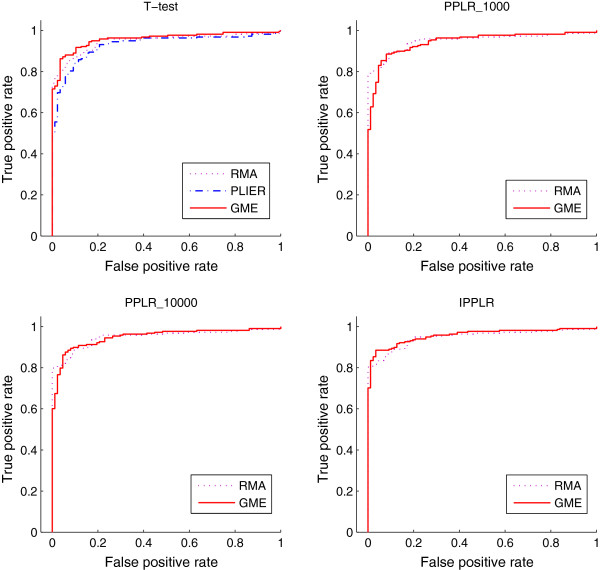
**ROC curves from different methods for 5-replicate Exon array data.** Gene expression estimation methods are combined with different finding-DE-gene methods. PLIER provides only a point estimate for gene expression and therefore is not applicable to PPLR and IPPLR. The number after PPLR indicates the sample number used in the importance sampling of the algorithm.

To evaluate the accuracy of GME for gene expression estimation from exon array data, we compare GME with the other two traditional methods RMA and PLIER. The functions implemented in Bioconductor package *affy* for RMA and PLIER methods are used to produce gene expression. We combine the different expression estimation methods with three DE detection methods, *t*-test, PPLR and IPPLR, to find DE genes on the MAQC dataset. *t*-test is applied to point estimates of gene expression from the three expression estimation methods. PPLR and IPPLR require a level of uncertainty associated with expression estimates, and they are therefore applied to GME and RMA which are able to provide expression measurement error. In addition to process all five replicates for each sample, we also randomly select two replicates to show the performance of each method with fewer number of replicates available. we repeat five runs for the processing of the 2-replicate case. Figure 2 shows the average ROC curves of the comparison for 2-replicate case and Figure 3 shows the results for 5-replicate case. GME combined with PPLR obtains lower true positive rate (TPR) at the top of ranking list of DE genes. However, by increasing the number of sample in the importance sampling of PPLR, TPR gets obviously improved. The area under ROC curve (AUC) for the different expression estimation methods combined with various DE detection methods are shown in Table 2. We can see from Table 2 that GME outperforms the other alternatives at most cases, especially when combined with *t*-test and IPPLR. The comparison results show that GME is a competitive approach in gene expression calculation from Exon array data.

**Table 2:** Area under ROC curves from different methods for Exon array data

**Methods**		**2 replicates**						**5 replicates**
		**1**	**2**	**3**	**4**	**5**	**Average**	
*t*-test	RMA	0.8945	0.8909	0.9107	0.9346	0.9316	0.9118	0.9475
	PLIER	0.8806	0.8852	0.9004	0.9084	0.9083	0.8937	0.9291
	GME	0.9082	0.9044	0.9415	0.9544	0.9427	0.9287	0.9580
PPLR_1000	RMA	0.9243	0.9234	0.9385	0.9417	0.9387	0.9323	0.9489
	GME	0.9208	0.9093	0.9365	0.9297	0.8969	0.9188	0.9447
*PPLR_10000	RMA	0.9227	0.9226	0.9419	0.9453	0.9432	0.9348	0.9492
	GME	0.9353	0.9317	**0.9474**	0.9374	0.9324	0.9274	0.9503
IPPLR	RMA	0.9246	0.9301	0.9464	0.9468	0.9463	0.9382	0.9493
	GME	**0.9379**	**0.9391**	0.9457	**0.9597**	**0.9549**	**0.9475**	**0.9589**

Gene expression estimation methods are combined with different finding-DE-gene methods. PPLR and IPPLR require a level of uncertainty associated with expression estimation, and they are therefore combined with GME and RMA since these two methods can provide variance of gene expression measurements. For *t*-test we use only the point estimates of gene expression. PLIER provides only a point estimate for gene expression and we only evaluate it combining with *t*-test. The number after PPLR indicates the sample number used in the importance sampling of the algorithm. The best result for each comparison is highlighted in bold.

### Validation of isoform expression estimation

**Figure 4:**
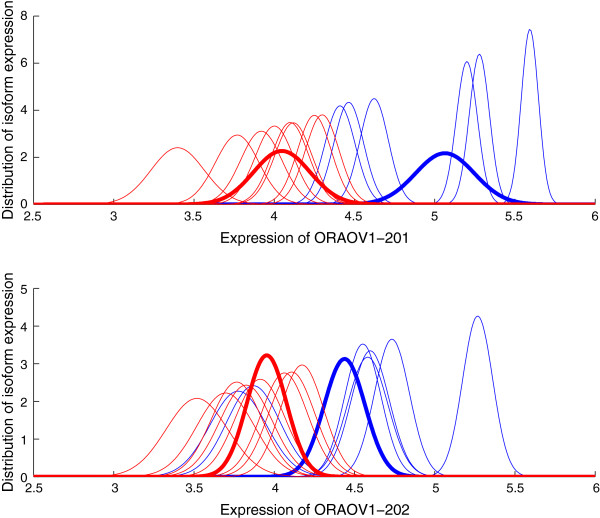
**Distribution of isoform expression for gene ORAOV1.** The distributions of the estimated isoform expression for the two alternatively spliced transcripts of gene ORAOV1 in the 15 cell lines are calculated from GME. The blue lines are for 11q13+ group and red lines for 11q13- group. The bold lines are the distributions of the mean expression for each group, obtained from PPLR. Expression is on the log scale.

**Figure 5:**
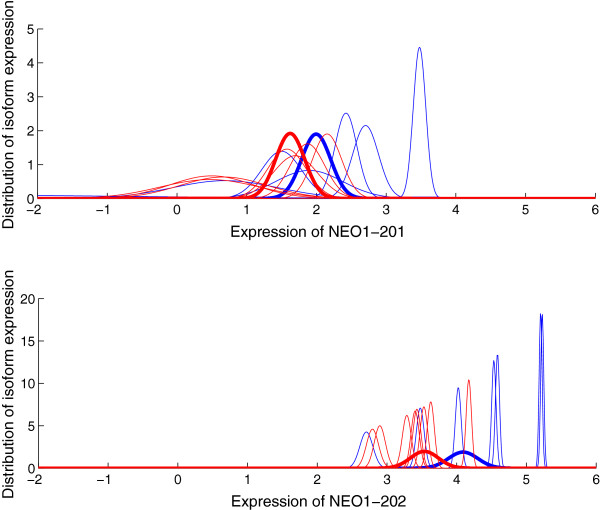
**Distribution of isoform expression for gene NEO1.** The distributions of the estimated isoform expression for the two alternatively spliced transcripts of gene NEO1 in the 15 cell lines are calculated from GME. The blue lines are for 11q13+ group and red lines for 11q13- group. The bold lines are the distributions of the mean expression for each group, obtained from PPLR. Expression is on the log scale.

We use the qRT-PCR validated HNSCC data set to verify the isoform expression calculated by GME. In HNSCC dataset, two ORAOV1 alternative splice variants (ORAOV1-201 and ORAOV1-202) and two NEO1 alternative splice variants (NEO1-201 and NEO1-202) are validated by qRT-PCR experiments. We apply GME to this dataset and obtain the expression levels for the four transcripts. For each transcript in every one of the 15 cell lines, GME produces the expression estimate and a level of uncertainty associated with this estimate. Figures 4 and 5 show the distributions of isoform expression in each cell line of ORAOV1 and NEO1, respectively. The blue lines are for 11q13+ samples and the red lines for 11q13- samples. We can see from the figures that there is considerable variability in the transcript expression for the cell lines from each sample group. High expression is generally associated with low variance while low expression with large variance. For the expression distribution of NEO1-201 as shown in the upper plot of Figure 5, there is extreme low expression for one cell line from each of the two sample groups. We then apply PPLR to the distributions of isoform expression to obtain the distributions of mean expression for each sample group, which are represented by the bold lines as shown in the figures. Note that the effects of low expression outliers are reduced by applying PPLR which accounts for technical and biological components of variance.

According to the qRT-PCR results, the four transcripts are overexpressed in 11q13+ sample with less significant change for ORAOV1-202 (*p*<0.0837). ORAOV1-201 presents higher expression levels than ORAOV1-202 in both 11q13+ and 11q13- samples, while NEO1-202 is expressed at higher levels than NEO1-201 in the two samples. Table 3 shows the directions of the relative expression change found by qRT-PCR and GME. The results “+” and “-” stand for up- and down-regulation in the first comparison component, respectively. For GME, the result of “+” indicates *PPLR*>0.5 and the result of “-” indicates *PPLR*<0.5. We also show the probability of differential expression as calculated by *max*(*PPLR*,1−*PPLR*), with numbers close to 1.0 indicating strong support. It can been seen from Table 3 that the relative expression changes found by GME combined with PPLR are consistent with qRT-PCR results for all comparisons. The results show that GME produces reliable isoform expression estimations for this specific dataset.

**Table 3:** GME results for the qRT-PCR validated transcripts

	Comparisons	**qRT-PCR**	**GME**	***m a x *( *P P L R *,1− *P P L R *)**	**Consistency**
11q13+ vs. 11q13-	ORAOV1-201	+	+	1.0000	Y
	ORAOV1-202	+	+	0.9968	Y
	NEO1-201	+	+	0.8961	Y
	NEO1-202	+	+	0.9719	Y
ORAOV1-201 vs. 202	11q13+	+	+	0.9154	Y
	11q13-	+	+	0.5782	Y
NEO1-201 vs. 202	11q13+	-	-	0.9999	Y
	11q13-	-	-	1.0000	Y

The expression changes between groups 11q13+ and 11q13- for each transcript, and between two transcripts of the same gene for each group, are examined. The results of qRT-PCR and GME are “+” or “-” for up- and down-regulation in the first comparison component, respectively. Column of max(PPLR,1-PPLR) gives the probability of differential expression. The concordances between qRT-PCR validation and GME results are given in the right-most column.

### Improvements for detection of differential expression

IPPLR accelerates the computation of PPLR by eliminating the importance sampling stage of the algorithm which significantly slows down PPLR computation. Table 4 shows the CPU run time of PPLR and IPPLR on 2-replicate and 5-replicate exon array data. The run time for 2-replicate data is the average processing time over the 5 runs. It can be seen from Table 4 that the computation time of PPLR increases with the number of importance samples and IPPLR is therefore much more computationally efficient. The accuracy of detecting DE genes for different methods is shown in Table 2. We can see that with the same expression estimation method, IPPLR obtains the best accuracy for most datasets. PPLR and IPPLR outperform *t*-test. PPLR was compared with more sophisticated moderated t-tests in the original publication [5]. These show the usefulness of measurement error propagated into the downstream analysis. The improvement is especially significant for the 2-replicate case demonstrating that probe-level measurement error helps to alleviate the need for experiment replicates. Note that as the number of importance samples increases the accurate of PPLR also gets improved. When the number of importance samples used is 10,000 then the accuracy of PPLR is close to that of IPPLR.

**Table 4:** Run time of PPLR and IPPLR

**Datasets**	**PPLR_1000**	**PPLR_10000**	**IPPLR**
2 replicates	73.1	1330.8	27.5
5 replicates	125.4	3127.4	15.9

The run time (CPU seconds) for 2-replicate dataset is the average processing time over the 5 runs. The number after PPLR indicates the sample number used in the importance sampling of the algorithm. The program runs on the machine with Intel Pentium Dual-core 2.6GHz CPU and 8.0G RAM.

### Accuracy of gene expression estimation for 3’ GeneChips

**Figure 6:**
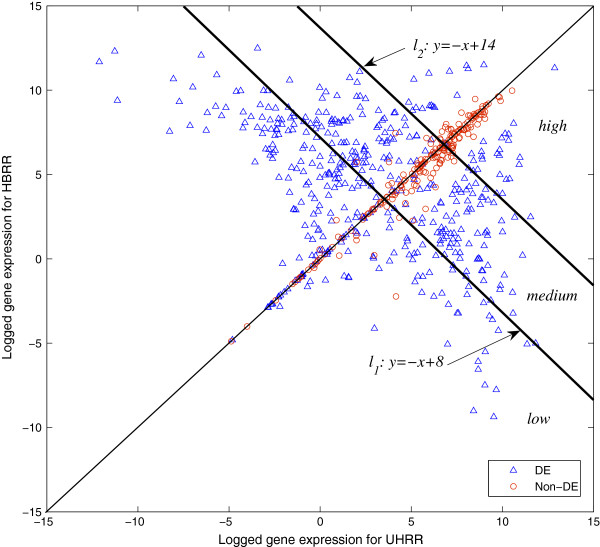
**The partition of qRT-PCR validated probe-sets in H133 GeneChip dataset.** Gene expression estimates are calculated from multi-mgMOS. The scatter plot is drawn with expression of HBRR sample against UHRR sample. Line *l*_1_:*y*=−*x*+8 and line *l*_2_:*y*=−*x*+14 partition the 656 qRT-PCR validated probe-sets into 3 groups, labelled as “low”, “median” and “high”.

**Figure 7:**
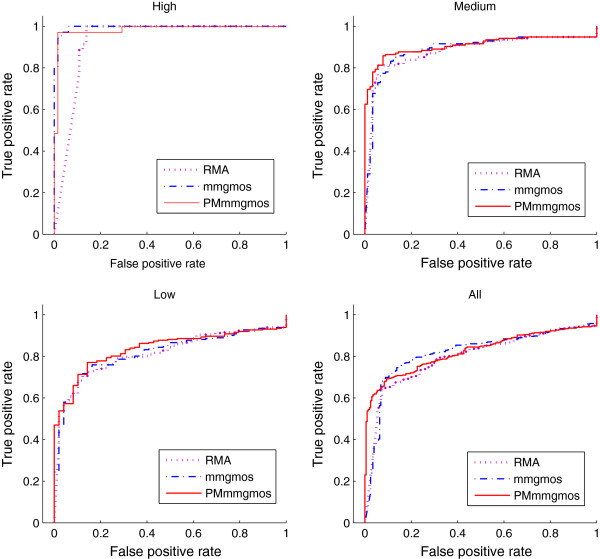
**ROC curves from different methods for U133 GeneChip data.** ROC curves are calculated from different gene expression estimation methods, RMA, multi-mgMOS and PM-only multi-mgMOS, combined with PPLR for “low”, “median”, “high” and “all” groups of U133 GeneChips data.

Our previous study [3] shows that the original multi-mgMOS presents good sensitivity to the concentration change in samples due to the correction of non-specific hybridisation by MM probe intensities. However, for weakly expressed genes the resulting logarithmic expression estimates are usually associated with large variance and this can cause instability in the downstream analysis. We divide the experimental data of Affymetrix U133 GeneChips into three groups, with “low”, “medium” and “high” expression respectively, to show this effect. Figure 6 shows the partition of the dataset with gene expression calculated from multi-mgMOS. Genes under line *l*_1_ belong to “low” expression group. Genes between line *l*_1_ and *l*_2_ belong to “median” expression group. Genes above line *l*_2_ belong to “high” expression group. The group of all genes is denoted as “all”. For each gene group, we plot ROC curves individually with the calculation from different expression methods combined with PPLR, as shown in Figure 7. The corresponding AUC values are shown in Table 5. We compare three expression estimation methods, PM-only multi-mgMOS, multi-mgMOS and the popular RMA approach. We can see that PM-only multi-mgMOS and multi-mgMOS outperform RMA for all gene groups. PM-only multi-mgMOS obtains better results than multi-mgMOS for “medium”, “low” and “all” groups, but fails in “high” group compared with multi-mgMOS. This shows PM-only multi-mgMOS performs better for relatively low expression genes while multi-mgMOS works well for high expression genes.

**Table 5:** Area under ROC curves from different methods for U133 GeneChip data

**Groups**	**# of probe-sets**			**PM-only multi-mgMOS**	**multi-mgMOS**	**RMA**
	**non-DE**	**DE+**	**DE-**			
High	65	21	14	0.9842	**0.9952**	0.9308
Medium	90	73	82	**0.9062**	0.8880	0.8827
Low	49	91	171	**0.8363**	0.8180	0.8147
All	204	185	267	**0.8227**	0.8130	0.7971

Genes are divided into three groups, labelled as “high”, “medium” and “low”, according to the expression levels. The numbers of “non-DE”, “DE+” and “DE-” probe-sets are shown. AUC is calculated individually for each of the three groups from PM-only multi-mgMOS, multi-mgMOS and RMA combined with PPLR. The overall AUC is also shown in the bottom of the table. The winner is highlighted in bold for each group.

**Figure 8:**
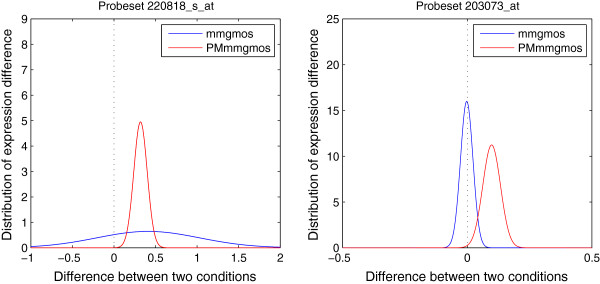
**Distribution of expression difference between two conditions for U133 GeneChip data.** Probe-set 220818_s_at is a low expression DE gene and probe-set 203073_at is a relatively highly expressed non-DE gene. The blue lines stand for the distributions of expression difference between two conditions calculated from multi-mgMOS and the red lines for PM-only multi-mgMOS.

We randomly select two probe-sets, 220818_s_at and 203073_at, out of probe-sets whose PPLR values are significantly different between multi-mgMOS and PM-only multi-mgMOS. Probe-set 220818_s_at is related to a low expression DE gene and 203073_at related to a high expression non-DE gene. The distributions of the expression difference between two conditions for the two probe-sets are shown in Figure 8. For the DE probe-set in the left plot, the two methods obtain similar mean values of the expression difference, but obviously different measurement error. The variance of the expression difference calculated from multi-mgMOS is much larger than PM-only multi-mgMOS and this results in lower PPLR value, 0.747, compared with 1.000 from PM-only multi-mgMOS (PPLR values close to 0 or 1 indicate significant DE). Thus, this probe-set is correctly classified as significant DE according to PM-only multi-mgMOS’s result while misclassified as non-DE according to multi-mgMOS’s computation. This shows that PM-only multi-mgMOS increases the stability of multi-mgMOS for gene expression calculation for lower expression. For the non-DE probe-set on the right plot of Figure 8, multi-mgMOS correctly classifies this probe-set with PPLR value 0.467 while PM-only multi-mgMOS misclassifies it with PPLR value 0.997 showing that PM-only multi-mgMOS can be less accurate in the high end.

### Robust clustering considering technical and biological variance

PUMA-CLUSTII is a robust Student’s *t* mixture model and takes into accounts expression measurement error, and technical and biological variance. Our work in [20] has already demonstrated that PUMA-CLUSTII obtained more accurate partitions compared with other alternatives on synthetic data. Furthermore, the method was shown to obtain numbers of clusters similar to the number of underlying groups in realistic simulated data. Applications of PUMA-CLUSTII on yeast metabolic cycle and cell cycle datasets have already shown that the method led to more biologically relevant clusters in terms of both GO category and TF-gene interaction.

## Conclusions

We have presented the extended and improved functions of the new version of the *puma* package and demonstrated the usefulness of these new functions on the well studied MAQC dataset and the qRT-PCR validated HNSCC dataset. With these extensions and improvements, *puma* is able to provide accurate expression estimates for both Affymetrix 3’ GeneChips and Exon arrays. In addition to gene expression measurements, the new *puma* can also provide reliable estimation of isoform expression from Exon array data. For 3’ GeneChip data, the stability of expression measurements for low expression genes was improved. Furthermore, a level of uncertainty associated with these expression estimates can also be obtained and this measurement error can be propagated into our downstream analysis approaches to obtain improved results. With the consideration of expression measurement error in the downstream analyses, methods can be computationally demanding. The new *puma* package significantly improves the computational efficiency of the previous method for finding DE genes and obtains even better accuracy. As the final contribution, the new *puma* provides a robust clustering method which considers the within-chip measurement error and across-chip technical and biological variance.

There are two main advantages of the new *puma* package. One is that the package processes Affymetrix 3’ GeneChips and Exon arrays to obtain accurate gene and isoform expression estimates with a level of uncertainty associated with these measurements. The other is that the package offers various downstream analysis approaches which make use of measurement error of expression to produce improved results at both gene and isoform level. Note that the data used for these downstream analyses is not limited to expression measurements from microarrays. The data can be expression measurement obtained from any other platform so long as a reasonable level of uncertainty can be associated with each measurement. For example, RNA-Seq is increasingly applied for transcript quantification [24]. Some methods proposed to analyse RNA-Seq data are able to provide both expression estimates and measurement uncertainty [25,26]. The transcript expression estimates and the related measurement error output by these methods can be used directly by the downstream analysis methods of *puma*. For all these reasons, *puma* is very useful to a large number of researchers who are interested in gene and transcript expression analysis.

## Methods

### Gamma model for Affymetrix GeneChip Exon array data

Let *y*_*gjc*_ represent the *j*th PM probe intensity for the *g*th gene under the *c*th condition. Allowing any number of isoform contributions to *y*_*gjc*_, we assume *y*_*gjc*_=*Σ*_*k*∈*M*(*gj*)_*s*_*gjkc*_, where *M*(*gj*) is the set containing indices of isoforms mapping to probe *j* of gene *g*, and *s*_*gjkc*_ is the intensity contribution from the *k*th mapping isoform. Similar to the assumption of the multi-mgMOS method for 3’ array, we assume *s*_*gjkc*_ follow a gamma distribution, *s*_*gjkc*_∼Ga(*α*_*gkc*_,*β*_*gj*_), where *β*_*gj*_ is a probe-specific latent variable which models the probe effects and is shared across the isoforms and experimental conditions of the same gene. As the summation of independent gamma-distributed variables, *y*_*gjc*_ also follows a gamma distribution, *y*_*gjc*_∼Ga(*Σ*_*k*∈*M*(*gj*)_*α*_*gkc*_,*β*_*gj*_). With a gamma prior for the latent variable *β*_*gj*_, i.e. *β*_*gj*_∼Ga(*c*_*g*_,*d*_*g*_), the likelihood of probe intensities for a specific gene is

(1) $$\begin{array}{lll} & L\left(\{y_{\text{gjc}}\}|\{\alpha_{\text{gkc}}\},c_{g},d_{g}\right)\; & \;\\ & \;\;=\underset{\text{jc}}{\prod }\int p(y_{\text{gjc}}|\Sigma_{k\in M(\text{gj})}\alpha_{\text{gkc}},\beta_{\text{gj}})p(\beta_{\text{gj}}|c_{g},d_{g})d\beta_{\text{gj}}.\; \end{array}$$

The integral in equation (1) can be computed analytically. The Maximum a Posteriori (MAP) solution of the model can thus be found by efficient numerical optimisation. With the estimated parameters $\left\{{\hat{\alpha }}_{\text{gkc}}\right\}$, ${ĉ}_{g}$ and ${\hat{d}}_{g}$, the distribution of the expression for each isoform is

(2) $$p(s_{\text{gjkc}})=\int p(s_{\text{gjkc}}|{\hat{\alpha }}_{\text{gkc}},\beta_{\text{gj}})p(\beta_{\text{gj}}|{ĉ}_{g},{\hat{d}}_{g})d\beta_{\text{gj}}.$$

We assume the expression of gene *g* is the sum of signal from its isoforms, i.e. *Σ*_*k*_*s*_*gjkc*_. Hence, the distribution of gene expression is also a gamma, *Σ*_*k*_*s*_*gjkc*_∼Ga(*Σ*_*k*_*α*_*gkc*_,*β*_*gj*_). Similarly, the posterior distribution of the gene expression can be expressed as

(3) $$p(\Sigma_{k}s_{\text{gjkc}})=\int p(\Sigma_{k}s_{\text{gjkc}}|\Sigma_{k}{\hat{\alpha }}_{\text{gkc}},\beta_{\text{gj}})p(\beta_{\text{gj}}|{ĉ}_{g},{\hat{d}}_{g})d\beta_{\text{gj}}.$$

The posterior distributions of the logged gene/isoform expression can be estimated from equation (2) and (3), respectively. The expectation of the logged expression level is then computed and approximated by a Gaussian. The Gaussian approximation to the posterior distribution is useful for propagating the probe-level measurement error in subsequent downstream analyses of both gene and isoform expression.

### PM-only multi-mgMOS for Affymetrix 3’ GeneChip data

Affymetrix 3’ GeneChips group probes into probe-sets. Most genes are covered by one probe-set and gene expression level can be presented by the expression estimated from the grouped probe intensities. To improve the stability of gene expression measurements for the original multi-mgMOS [3], we ignore the MM probe signal and assume PM probes measure specific hybridisation in a probe-specific way. The intensities of PM probes within a probe-set are assumed to follow a gamma distribution. Let *y*_*ijc*_ represent the *j*th PM intensity for the *i*th probe-set under the *c*th condition. The model is defined by

(4) $$y_{\text{ijc}}\;∼\;\text{Ga}(\alpha_{\text{ic}},b_{\text{ij}})$$

(5) $$b_{\text{ij}}\;∼\;\text{Ga}(c_{i},d_{i}),$$

where *b*_*ij*_ is a latent variable which models probe-specific effects for the same type of chip.

The MAP solution of this model can be easily found by efficient numerical optimisation. With the estimated parameters ${\hat{\alpha }}_{\text{ic}}$, ${ĉ}_{i}$ and ${\hat{d}}_{i}$, the posterior distribution of PM intensities is

(6) $$P(y_{\text{ijc}})=\int P(y_{\text{ijc}}|{\hat{\alpha }}_{\text{ic}},b_{\text{ij}})P(b_{\text{ij}}|{ĉ}_{i},{\hat{d}}_{i})db_{\text{ij}}.$$

We use a Gaussian with a mean ${\hat{\mu }}_{\text{ic}}$ and a variance ${\hat{\sigma }}_{\text{ic}}$ to approximate the posterior distribution of the expectation of log(*y*_*ijc*_). The mean of the Gaussian is taken as the estimated gene expression and the variance shows the measurement error associated with this estimate.

### Improved PPLR for finding differential expressed genes

In order to overcome the computation limitation of the original PPLR model, we propose an improved PPLR model (IPPLR) to detect DE genes. Similar to PPLR, IPPLR also considers both expression estimates and measurement uncertainty to obtain high accuracy in finding DE genes. We add a hidden variable *x*_*ij*_ to the original PPLR model, representing the true gene expression. We assume that the variable is Gaussian distributed $x_{\text{ij}}∼N(\mu_{j},\lambda^{-1})$, where *μ*_*j*_ is the mean logged expression level under condition *j* and *λ* is the inverse of the between-replicate variance and is shared across different conditions. The measured expression level ${\hat{x}}_{\text{ij}}$ can be expressed as,

(7) $${\hat{x}}_{\text{ij}}∼N(x_{\text{ij}},s_{\text{ij}}^{2}),$$

where $s_{\text{ij}}^{2}$ is the probe-level measurement error, which can be obtained from multi-mgMOS or PM-only multi-mgMOS.

We make a prior assumption that *μ*_*j*_ and *λ*^−1^ are independent and put a Gaussian prior on *μ*_*j*_,

(8) $$\mu_{j}∼N(\mu_{0},\eta_{0}^{-1}),$$

where *μ*_0_ and *η*_0_ are hyperparameters, on which we adopt noninformative hyperpriors. We assume a conjugate gamma prior on *λ*,

(9) λ∼Ga(α,β).

We use the EM algorithm combined with a variational method to work out the model. In the E-step of PPLR, the variational distribution of *λ* is obtained by importance sampling which slows down the computation of the method. In contrast, the computation in the E-step of IPPLR is analytical due to the introduction of the latent variable *x*_*ij*_. IPPLR is therefore more computationally efficient than PPLR.

Once the posterior distribution of *μ*_*j*_ is obtained, the probability of positive log-ratio (PPLR) between a treatment *μ*_*t*_ and a control *μ*_*c*_ can be calculated by

(10) $$\text{PPLR}=\overset{+\infty }{\underset{0}{\int }}d(\mu_{t}-\mu_{c})P(\mu_{t}-\mu_{c}|D,\hat{\phi }),$$

where *D* is the observed dataset and $\hat{\phi }$ is the set of ML estimates of hyperparameters. The examined transcript is up-regulated in the treatment when *PPLR*>0.5 while down-regulated when *PPLR*<0.5.

### PUMA-CLUSTII for clustering of replicated gene expression

For the cases where technical or biological replicates are available, we propose a robust Student’s *t*-mixture model to deal with the technical and biological variability. Suppose the expression estimate for gene *n* under condition *j* is *x*_*nji*_, and the corresponding true expression and the known probe-level measurement error are *t*_*nji*_ and *s*_*nji*_ respectively, where *i*=1,…,*R*_*j*_ and *R*_*j*_ is the number of replicates under condition *j*. The expression estimate *x*_*nji*_ is assumed to be generated from the following Gaussian distribution,

(11) $$x_{\text{nji}}∼N(t_{\text{nji}},s_{\text{nji}}).$$

The true gene expression *t*_*nji*_’s for the replicates under the same condition is also assumed to be drawn from a Gaussian distribution,

(12) $$t_{\text{nji}}∼N\left(w_{\text{nj}},\frac{1}{\eta_{n}}\right),$$

with the mean expression *w*_*nj*_ for condition *j* and the precision *η*_*n*_. By introducing a latent variable *u*_*n*_ for each gene, the *t*-distribution can be written as a convolution of a Gaussian with a Gamma placed on its precisions,

(13) $$\;\text{St}\left(w_{n}|\mu_{k},\Sigma_{k},\nu_{k}\right)=\overset{\infty }{\underset{0}{\int }}N\left(w_{n}|\mu_{k},\frac{\Sigma_{k}}{u_{n}}\right)\text{Ga}\left(u_{n}|\frac{\nu_{k}}{2},\frac{\nu_{k}}{2}\right)\text{du},$$

where *μ*_*k*_ and *Σ*_*k*_ denote the mean and covariance matrix, respectively, and *ν*_*k*_ is degrees of freedom, for component *k*. The mean expression vector *w*_*n*_ is modelled as a robust mixture of Student’s *t*-distributions.

(14) $$p(w_{n})=\overset{K}{\underset{k=1}{\sum }}\Pi_{k}\text{St}(w_{n}|\mu_{k},\Sigma_{k},\nu_{k}).$$

We share *η*_*n*_ across all conditions for each gene and assume that it captures the biological gene-specific variability. The precision *η*_*n*_ is assumed to come from a Gamma distribution

(15) $$\eta_{n}|z_{\text{nk}}=1∼\text{Ga}(\alpha_{k},\beta_{k}).$$

Inference can be carried out using the variational EM algorithm. Specifying the maximum and minimum numbers of components, the algorithm automatically converged to the optimal number of mixture components by employing the minimum message length (MML) principle [27] for model selection.

## Availability and requirements

**Project name:** puma Software

**Project home page:**http://www.bioinf.manchester.ac.uk/resources/puma

**Operating systems:** Platform independent

**Programming language:** R, C

**Other requirements:** R

**Any restrictions to use:** it is available for free download except that puma uses C scripts of donlp [28].

## Competing interests

The authors declare that they have no competing interests.

## Authors’ contributions

XL developed PUMA-CLUSTII, partially supervised the development of the extensions of the *puma* package and wrote the manuscript. ZG developed GME and PM-only multi-mgMOS methods. LZ developed IPPLR method. MR initiated the puma project and partially supervised the development of the *puma* package. All authors read and approved the final manuscript.

## References

[B1] Łabaj PP Leparc GG E LB Markillie LM S WH P KD Characterization and improvement of RNA-Seq precision in quantitative transcript expression profiling Bioinformatics 2011 27 13 i383 i391 10.1093/bioinformatics/btr247 21685096 PMC3117338

[B2] Pearson RD Liu X Sanguinetti G Milo M D LN Rattray M puma: a bioconductor package for propagating uncertainty in microarray analysis BMC Bioinformatics 2009 10 211 10.1186/1471-2105-10-211 19589155 PMC2714555

[B3] Liu X Milo M Lawrence ND Rattray M A tractable probabilistic model for Affymetrix probe-level analysis across multiple chips Bioinformatics 2005 21 3637 3644 10.1093/bioinformatics/bti583 16020470

[B4] Sanguinetti G MIlo M Rattray M Lawrence ND Accounting for probe-level noise in principal component analysis of mmicroarray data Bioinformatice 2005 21 3748 3754 10.1093/bioinformatics/bti617 16091409

[B5] Liu X Milo M Lawrence ND Rattray M Probe-level measurement error improves accuracy in detecting differential gene expression Bioinformatics 2006 22 2107 2113 10.1093/bioinformatics/btl361 16820429

[B6] Liu X Lin KK Andersen B Rattray M Including probe-level uncertainty in model-based gene expression clustering BMC Bioinformatics 2007 9 98 17376221 10.1186/1471-2105-8-98 PMC1847531

[B7] Affymetrix Guide to Probe Logarithmic Intensity Error 2008 [Technical note]

[B8] Irizarry RA Hobbs B Collin F Beazer-Barclay YD Antonellis KJ Exploreation, normalization, and summaries of high density oligonucleotide array probe level data Biostatistics 2003 4 249 264 10.1093/biostatistics/4.2.249 12925520

[B9] Affymetrix Alternative Transcript Analysis Methods for Exon Arrays 2005 (11 October 2005, date last revised) [Http://media.affymetrix.com/support/technical/whitepapers/exon_alt_transcript_analysis_whitepaper.pdf]

[B10] Purdom E Simpson KM Robinson MD Conboy JG Lapuk AV Speed TP FIRMA: a method for detection of alternative splicing from exon array data Bioinformatics 2008 24 1707 1714 10.1093/bioinformatics/btn284 18573797 PMC2638867

[B11] Xing Y Stoilov P Kapur K Han A Jiang H Shen S Black DL Wong WH MADS: a new and improved method for analysis of differential alternative splicing by exon-tiling microarrays RNA 2008 14 1470 1479 10.1261/rna.1070208 18566192 PMC2491471

[B12] Risueño A Fontanillo C E DM J DLR GATExplorer: genomic and transcriptomic explorer; mapping expression probe to gene loci, transcripts, exons and ncRNAs BMC Bioinformatics 2010 11 221 10.1186/1471-2105-11-221 20429936 PMC2875241

[B13] Wu Z Irizarry RA Gentleman R Martinez-Murillo F Spencer F A model-based background adjustment for oligonucleotide expression arrays J Am Stat Assoc 2004 99 909 917 10.1198/016214504000000683

[B14] Turro E Lewin A Rose A Dallman MJ Richardson S MMBGX: a method for estimating expression at the isoform level and detecting differential splicing using whole-transcript Affymetrix arrays Nucleic Acids Res 2010 38 e4 10.1093/nar/gkp853 19854940 PMC2800219

[B15] Chen P Lepikhova T Hu Y Monni O Hautamiemi S Comprehensive exon array data processing method for quantitative analysis of alternative spliced variants Nucleic Acids Res 2011 39 e123 10.1093/nar/gkr513 21745820 PMC3185423

[B16] Li C Wong W Model-based analysis of oligonucleotide arrays: Expression index computation and outlier detection Proc Natl Acad Sci USA 2001 98 31 36 10.1073/pnas.98.1.31 11134512 PMC14539

[B17] Bishop CM Pattern Recognition and Machine Learning 2006 New York: Springer

[B18] Pearson RD A comprehensive re-analysis of the Golden Spike data: Towards a benchmark for differential expression methods BMC Bioinformatice 2008 9 164 10.1186/1471-2105-9-164 PMC2324099 18366762

[B19] Zhang L Liu X An improved probabilistic model for finding differential gene expression Proceedings of the 2nd International Conference on BioMedical Engineering and Informatics, BMEI 2009 2009 Tianjin, China

[B20] Liu X Rattray M Including probe-level measurement error in robust mixture clustering of replicated microarray gene expression Stat Appl Genet Mol Biol 2010 9 42 10.2202/1544-6115.1600 21194414

[B21] Consortium M The MicroArray Quality Control (MAQC) project shows inter- and intraplatform reproducibility of gene expression measurements Nat Biotechnol 2006 24 1151 1161 10.1038/nbt1239 16964229 PMC3272078

[B22] Canales RD Luo Y Willey JC Austermiller B Barbacioru CC Boysen C Hunkapiller K Jensen RV Knight CR Y LK Ma Y Maqsodi B Papallo A Peters EH Poulter K L RP Samaha RR Shi L Yang W Zhang L M GF Evaluation of DNA microarray results with quantitative gene expression platforms Nat Biotechnol 2006 24 1115 1122 10.1038/nbt1236 16964225

[B23] Bullard JH Purdom E Hansen KD Dudoit S Evaluation of statistical methods for normalization and differential expression in mRNA-Seq experiments BMC Bioinformatics 2010 11 94 10.1186/1471-2105-11-94 20167110 PMC2838869

[B24] Nagalakshmi U Wang Z Waem K Shou C Raha D Gerstein M Snyder M The transcriptional lanscape of the yeast genome defined by RNA sequencing Science 2008 320 1344 1349 10.1126/science.1158441 18451266 PMC2951732

[B25] Katz Y Wang ET Airoldi EM Burge CB Analysis and design of RNA sequencing experiments for identifying isoform regulation Nat Methods 2010 7 1009 1015 10.1038/nmeth.1528 21057496 PMC3037023

[B26] Glaus P Honkela A Rattray M Identifying differentially expressed transcripts from RNA-seq data with biological variation Bioinformatics 2012 28 1721 1728 10.1093/bioinformatics/bts260 22563066 PMC3381971

[B27] Figueiredo MAT Jain AK Unsupervised learning of finite mixture models IEEE Trans Pattern Anal Mach Intell 2002 24 381 396

[B28] Spellucci PDB An SQP method for general nonlinear programs using only equality constrained subproblems Math Program 1998 82 413

